# Sudomotor Dysfunction as an Early Marker of Autonomic and Cardiovascular Risk in Diabetes: Insights from a Cross-Sectional Study Using SUDOSCAN

**DOI:** 10.3390/bios15060372

**Published:** 2025-06-10

**Authors:** Larisa Anghel, Claudiu Cobuz, Laura-Cătălina Benchea, Vasile Maciuc, Maricela Cobuz, Radu-Andy Sascău, Cristian Stătescu

**Affiliations:** 1 Internal Medicine Department, “Grigore T. Popa” University of Medicine and Pharmacy, 700503 Iași, Romania; larisa.anghel@umfiasi.ro (L.A.); benchea.laura-catalina@d.umfiasi.ro (L.-C.B.); radu.sascau@umfiasi.ro (R.-A.S.); cristian.statescu@umfiasi.ro (C.S.); 2 Cardiology Department, Cardiovascular Diseases Institute “Prof. Dr. George I. M. Georgescu”, 700503 Iași, Romania; 3 Faculty of Medicine and Biological Sciences, Stefan cel Mare University of Suceava, 720229 Suceava, Romania; 4 Department of Animal Resources and Technologies, Faculty of Food and Animal Sciences, “Ion Ionescu de la Brad” University of Life Sciences, 700489 Iasi, Romania; vmaciuc@uaiasi.ro; 5 “Sfântul Ioan cel Nou” Emergency Clinical Hospital, 720224 Suceava, Romania; cobuz.maricela@spjsv.ro

**Keywords:** diabetic autonomic neuropathy, sudomotor dysfunction, SUDOSCAN, cardiovascular risk

## Abstract

Background: Diabetic neuropathy, particularly in its autonomic form, is often underdiagnosed despite its clinical significance. Electrochemical skin conductance (ESC), measured by SUDOSCAN, offers a non-invasive way to assess the autonomic dysfunction. Methods: A total of 288 diabetic patients were assessed using SUDOSCAN to measure ESC in the hands and feet. Clinical and laboratory parameters, including glycated hemoglobin (HbA1c), body mass index (BMI), blood pressure, lipid profile, and cardiovascular risk, were analyzed for correlations with ESC. Neuropathy status was evaluated, and ROC analysis was performed to assess diagnostic accuracy. Results: Sudomotor dysfunction was prevalent, particularly in patients with a diabetes duration exceeding 20 years (*p* < 0.05). Men showed significantly higher right foot ESC than women (76.5 ± 13.1 vs. 74.0 ± 13.5 µS, *p* = 0.041). A strong inverse correlation was found between cardiovascular risk score and right foot ESC (r = −0.455, *p* < 0.001). Left foot ESC also correlated inversely with cardiovascular risk (r = −0.401, *p* < 0.001) and HbA1c (r = −0.150, *p* = 0.049), while a weak positive correlation was seen with BMI (r = 0.145, *p* = 0.043). ROC analysis showed the highest area under the curve (AUC) in right foot ESC for autonomic neuropathy (AUC = 0.750, 95% CI: 0.623–0.877, *p* < 0.001). Conclusions: This study is among the few to systematically correlate ESC with validated cardiovascular risk scores in a diabetic outpatient cohort, highlighting its potential as a novel early screening biomarker for autonomic and cardiovascular complications.

## 1. Introduction

Diabetes mellitus is a chronic metabolic disease characterized by hyperglycemia resulting from defects in insulin secretion, insulin action, or both. Globally, the prevalence of diabetes has reached epidemic proportions, with the number of affected individuals increasing from approximately 275 million in 2010 to over 537 million in 2021. Current projections estimate that this number will rise to 643 million by 2030 and 783 million by 2045 [1]. The burden of diabetes extends beyond glycemic dysregulation, as the disease is associated with a wide range of microvascular (neuropathy, retinopathy, nephropathy) and macrovascular (coronary artery disease, stroke, peripheral artery disease) complications that substantially impair quality of life and increase healthcare costs [2,3].

Among the microvascular complications, diabetic neuropathy (DN) stands out due to its high prevalence and multifactorial nature. It affects up to 60% of individuals with diabetes over the course of the disease and can manifest in both peripheral and autonomic forms [4,5]. While distal symmetric polyneuropathy is the most common presentation, autonomic neuropathy, particularly cardiac autonomic neuropathy (CAN), is often overlooked in clinical practice due to its insidious onset and nonspecific symptoms. Despite being less frequently diagnosed, CAN is associated with increased mortality and a higher risk of silent myocardial ischemia and sudden cardiac death [6].

Early detection of diabetic autonomic neuropathy is critical for timely intervention and prevention of progression. However, conventional diagnostic tools—such as quantitative sudomotor axon reflex testing (QSART), cardiovascular reflex tests, or nerve conduction studies—are often time-consuming, expensive, or require specialized personnel [2,3]. This limits their routine application in many outpatient or primary care settings. In this context, sudomotor function, mediated by unmyelinated sympathetic C-fibers, has emerged as a sensitive marker for early autonomic dysfunction [7].

To address these diagnostic challenges, the SUDOSCAN device has been developed as a non-invasive, quick, and user-friendly method for assessing electrochemical skin conductance (ESC). The SUDOSCAN device has been introduced as a novel tool for the early detection of diabetic autonomic neuropathy, particularly for screening cardiac autonomic neuropathy by evaluating sudomotor function [2,8,9]. The CAN risk score generated by SUDOSCAN incorporates electrochemical skin conductance along with patient-specific variables, such as age, height, weight, and glycated hemoglobin levels. This technology offers a practical alternative for screening diabetic neuropathy in everyday clinical settings [9,10,11]. Additionally, neuropathy is a major contributor to diabetic foot syndrome (DFS), a debilitating complication associated with significant morbidity and risk of amputation. The pathophysiology of DFS involves a combination of neuropathy, ischemia, and mechanical stress [3]. Sudomotor dysfunction contributes to skin dryness, impaired wound healing, and ulceration—highlighting the potential value of sudomotor assessment in the broader screening of high-risk patients, including those at risk for DFS [7].

Sudomotor dysfunction, indicative of small fiber neuropathy, is recognized as an early and quantifiable marker of autonomic involvement in diabetic patients. Since diabetes is the most widespread metabolic disorder globally and a key contributor to cardiovascular risk, assessing sudomotor function may help identify individuals at increased risk for both neuropathic and cardiovascular complications [6]. The SUDOSCAN device employed in this study has been specifically validated for detecting autonomic dysfunction in diabetic populations, reinforcing its clinical relevance.

This study is predicated on the premise that sudomotor dysfunction, as a manifestation of small fiber neuropathy, represents an accessible marker of systemic autonomic impairment in diabetes. The CAN risk score algorithm integrated within the SUDOSCAN device leverages electrochemical skin conductance alongside demographic and metabolic variables, providing a model-based rationale for its use in our analysis.

Our primary objective was to investigate the relationship between ESC, measured non-invasively using SUDOSCAN, and early indicators of autonomic and cardiovascular risk in a real-world outpatient cohort with diabetes. We assessed the prevalence of peripheral and autonomic neuropathy and examined associations between sudomotor function and key clinical and metabolic parameters, including HbA1c, BMI, and diabetes duration, to identify potential predictors of autonomic dysfunction. Additionally, we explored links between ESC and systemic diabetic complications, such as cardiovascular and renal risk scores, to evaluate the potential of SUDOSCAN as a practical early screening tool for multisystem involvement in diabetes. Thus, the rationale for this study arises from the clinical need to identify early, non-invasive markers of autonomic and cardiovascular risk in patients with diabetes, a population characterized by high morbidity and mortality due to diabetic neuropathy.

By integrating clinical, biochemical, and functional data, this study contributes to the growing body of evidence supporting the use of sudomotor testing as a practical tool for early detection and risk stratification in diabetic neuropathy.

## 2. Materials and Methods

### 2.1. Patients

This observational, cross-sectional study was conducted at Diabetes, Nutrition and Metabolic Diseases Outpatient Department of the “Sfântul Ioan cel Nou” Clinical Hospital in Suceava, Romania, between October 2024 and February 2025. Ethical approval was obtained from the host institution’s Ethics Committee (Approval No. 20/24 April 2024), and all participants provided written informed consent prior to enrollment.

This study included 288 adult patients diagnosed with type 1 or type 2 diabetes mellitus, with or without symptoms suggestive of diabetic neuropathy. The exclusion criteria were pregnancy or lactation, limb amputation, presence of pacemakers or other implantable medical devices, open or infected wounds on the hands or feet, and a history of epilepsy or active seizure disorders.

All participants underwent a standardized medical examination conducted by trained physicians. Data collected included age, sex, diabetes type and duration, weight, height, body mass index, resting systolic and diastolic blood pressure (SBP/DBP), and current treatments (including neurotrophic medication). Blood pressure was recorded after 5 min of rest in the supine position.

Laboratory values, such as glycated hemoglobin, total cholesterol, low density lipoprotein cholesterol (LDLc), triglycerides, and renal function indicators, were obtained from recent medical records or measured using certified clinical analyzers from Diabetes, Nutrition and Metabolic Diseases Outpatient Department of the “Sfântul Ioan cel Nou” Clinical Hospital in Suceava, Romania. The HbA1c level was used as a marker of long-term glycemic control.

### 2.2. Assessment of Sudomotor Function

Sudomotor function was assessed using the SUDOSCAN device (Impeto Medical, Paris, France), a non-invasive system based on reverse iontophoresis and chronoamperometry. This method has been validated in various clinical studies [7,9,11,12]. Patients were instructed to avoid the use of lotions, creams, or emollients on their hands or feet prior to the test to ensure optimal skin–electrode conductivity. Measurements were taken in a temperature-controlled setting.

Electrochemical skin conductance was measured using the SUDOSCAN device, which operates on the principle of reverse iontophoresis and chronoamperometry. During the test, patients placed their palms and soles on stainless steel electrodes, and a low-voltage direct current (1–4 V) was applied to stimulate chloride ion movement. The resulting current, indicative of sweat gland function and innervation by small unmyelinated sympathetic C-fibers, was used to calculate ESC in microSiemens (µS).

ESC reflects chloride ion flow, which is indicative of sweat gland function and its innervation by small unmyelinated sympathetic C-fibers. Based on validated thresholds, ESC values were interpreted as follows:

- Normal autonomic function: ESC > 60 μS (hands and feet);
- Possible autonomic neuropathy: ESC 40–60 μS;
- Confirmed/advanced autonomic neuropathy: ESC < 40 μS (either hand or foot).

The SUDOSCAN device incorporates proprietary algorithms that estimate the risk of cardiac autonomic neuropathy by integrating electrochemical skin conductance values with patient-specific variables, such as age, height, weight, and glycated hemoglobin. This results in a calculated CAN risk score, which categorizes individuals into the following risk levels: - low risk: <25%; - moderate risk: 25–49%; - high risk: ≥50%. Although not specific for large fiber dysfunction, it may also provide insight into early small fiber involvement in peripheral polyneuropathy.

### 2.3. Statistical Analysis

The statistical analysis was performed using IBM SPSS Statistics version 20.0 (IBM, New York, NY, USA). Descriptive statistics were used to characterize the study population. For continuous variables, the results were expressed as the mean ± standard deviation (SD) and standard error of the mean. The associations between quantitative variables were evaluated using either Pearson’s or Spearman’s correlation coefficients, depending on the distribution and scale of the data. For comparisons involving categorical variables, the chi-square test or Fisher’s exact test was employed, as appropriate. When analyzing differences between two groups based on continuous or ordinal variables, the Student’s *t*-test was used for normally distributed data, while the Mann–Whitney U test was applied for non-parametric distributions.

Linear and multiple linear regression models were used to explore the predictive relationship between sudomotor function (dependent variable) and clinical parameters, such as HbA1c, BMI, and disease duration. The ENTER method was employed in multiple regression to assess the independent contribution of each predictor.

Group comparisons were made using analysis of variance (ANOVA). When significant differences were found, Tukey’s honest significant difference (HSD) test was applied for post hoc pairwise comparisons to identify which groups differed significantly.

Receiver operating characteristic (ROC) curve analysis was performed to evaluate the diagnostic performance of SUDOSCAN and to determine its sensitivity and specificity in identifying patients at risk for autonomic neuropathy and related complications. The area under the curve was calculated to assess the overall accuracy of the test. The statistical significance level was set at *p* < 0.05, while values below *p* < 0.001 indicated high statistical significance.

## 3. Results

Among the 288 patients enrolled in this study, men (n = 156) and women (n = 132) were compared across key clinical and functional parameters.

### 3.1. Patient Characteristics

Men had a slightly higher mean age (59.3 ± 11.8 years) compared to women (57.6 ± 12.6 years). Regarding diabetes type, the majority of patients (83.2%) were diagnosed with type 2 diabetes mellitus (T2DM), while 16.8% had type 1 diabetes mellitus (T1DM). Also, women presented with a slightly higher mean BMI of 33.2 ± 16.9 kg/m^2^ compared to 31.8 ± 15.9 kg/m^2^ in men. HbA1c levels were relatively comparable between sexes, with men averaging 8.5 ± 2.0% and women averaging 8.3 ± 1.9%, indicating poor glycemic control across both groups. In terms of cardiovascular indicators, men exhibited slightly elevated systolic and diastolic blood pressures as well as higher average cardiovascular risk scores. The mean cardiovascular risk score was 33.6 ± 11.9 in men compared to 30.5 ± 12.5 in women.

Across all measurements of sudomotor function, men demonstrated marginally higher electrochemical skin conductance values than women. For instance, right foot conductivity averaged 76.5 ± 13.1 µS in men versus 74.0 ± 13.5 µS in women. Similarly, left hand ESC was 66.3 ± 14.4 µS in men and 64.7 ± 14.8 µS in women, suggesting a trend toward better sudomotor function in the male subgroup (Table 1). These findings suggest a statistically significant disparity in sudomotor function and systemic complication risk between male and female diabetic patients, underlining the potential role of gender in autonomic neuropathy expression and its associated risks. Further investigation may be warranted to explore the underlying mechanisms and implications for personalized diabetes care.

### 3.2. Correlations Between Sudomotor Function and Clinical Parameters

#### 3.2.1. Sudomotor Function and Cardiovascular Risk

**Figure 1:**
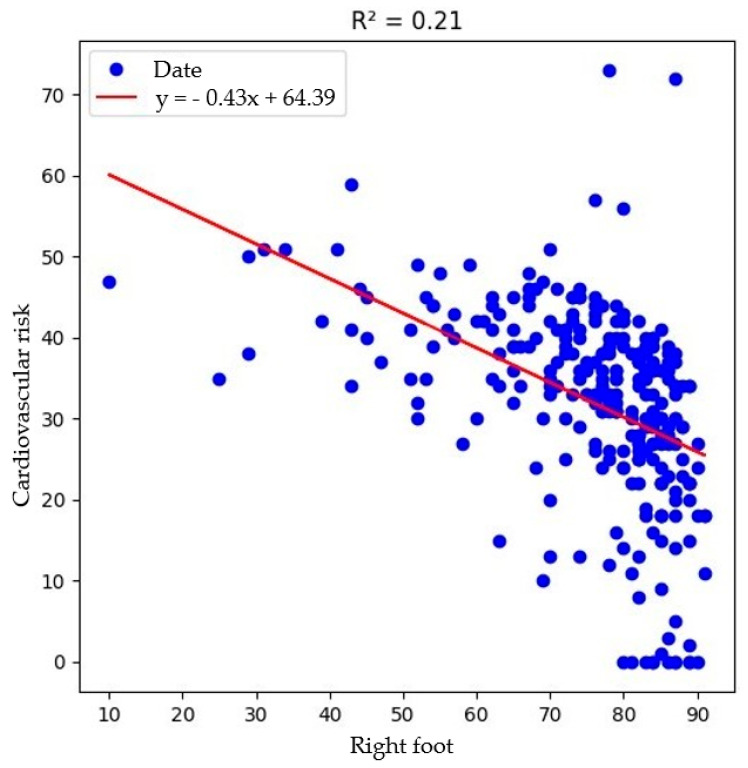
Highly significant inverse correlation between right foot electrochemical skin conductance and cardiovascular risk score.

A strong and highly significant inverse correlation was found between cardiovascular risk scores and right foot ESC (r = −0.455, *p* < 0.001), with a moderate inverse correlation observed for left foot ESC (r = −0.401, *p* < 0.001) (Figure 1). These results suggest that lower sudomotor activity is closely linked to elevated cardiovascular risk in diabetic patients. This association supports the hypothesis that autonomic dysfunction, particularly in the cardiovascular domain, may coexist with peripheral sudomotor impairment. The reduction in sweat gland innervation may reflect systemic autonomic and endothelial dysfunction, reinforcing the role of ESC as a non-invasive tool for cardiovascular risk stratification in diabetes care.

#### 3.2.2. Sudomotor Function and Glycemic Control

A weak but statistically significant inverse correlation was observed between HbA1c and left foot ESC (r = −0.150, *p* = 0.049). This finding indicates that poorer long-term glycemic control may contribute to sudomotor dysfunction, potentially via microvascular damage and small fiber neuropathy. While the correlation is modest, it aligns with existing literature suggesting that chronic hyperglycemia is a contributor to early autonomic nerve impairment.

#### 3.2.3. Sudomotor Function and Body Mass Index

Interestingly, a weak positive correlation was identified between BMI and left foot ESC (r = 0.145, *p* = 0.043). Although initially counterintuitive—given that obesity is generally linked to worsened autonomic function—this result may reflect confounding factors such as skin hydration or sweat gland responsiveness. Notably, this association was not observed in the hands or right foot and, therefore, should be interpreted cautiously.

While not all correlations were strong, the consistent and significant inverse associations between ESC values and cardiovascular risk—particularly in the lower extremities—underscore the utility of ESC measurement via SUDOSCAN in diabetes care. These findings support the inclusion of sudomotor testing in the broader assessment of systemic complications in diabetic patients.

**Figure 2:**
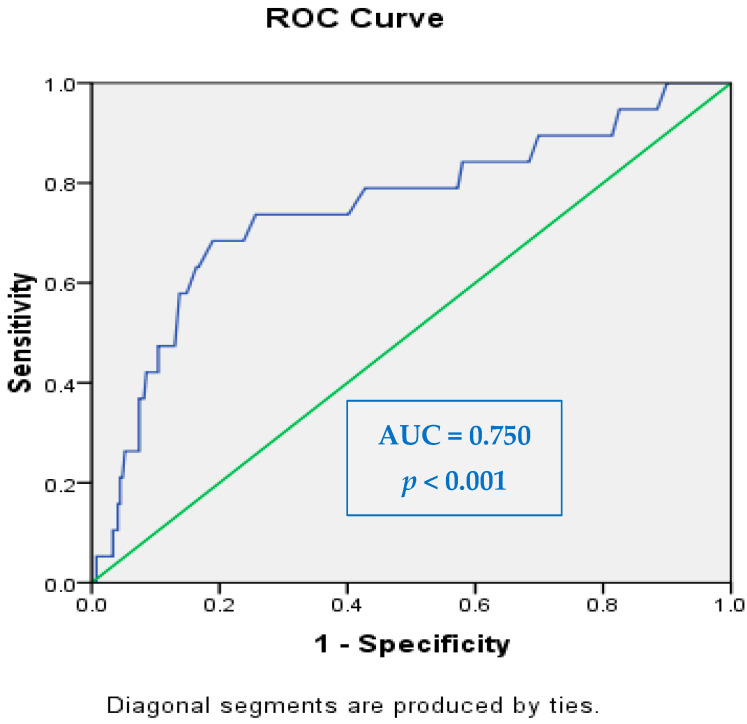
ROC curve for Sudoscan-derived electrochemical skin conductance in predicting autonomic neuropathy—highest diagnostic accuracy observed in right foot.

Electrochemical skin conductance values obtained via Sudoscan were analyzed in relation to the presence of peripheral neuropathy (PNP) and autonomic neuropathy. In both hands and feet, ESC values were significantly lower in patients with confirmed PNP and autonomic dysfunction, suggesting impaired sudomotor function. ROC curve analysis demonstrated moderate to higher discriminatory power, with area under the curve values ranging from 0.398 to 0.750 depending on the anatomical region and neuropathy type. The highest diagnostic accuracy was observed in right foot (AUC = 0.750, 95% CI [0.623–0.877], *p* < 0.001) for autonomic neuropathy (Figure 2), followed by left foot (AUC = 0.640, 95% CI [0.512–0.747], *p* = 0.021).

## 4. Discussion

In this study involving 288 patients with diabetes, we investigated the utility of electrochemical skin conductance, as measured by SUDOSCAN, in evaluating sudomotor dysfunction and its association with metabolic control, cardiovascular risk, and the presence of peripheral and autonomic neuropathies. The findings provide compelling evidence that sudomotor function assessment is a valuable tool in the early identification of diabetic complications, particularly autonomic neuropathy and systemic cardiovascular risk.

Consistent with existing literature, men demonstrated slightly better sudomotor function compared to women, as evidenced by higher ESC values across all anatomical regions. Although differences were modest, they reached statistical significance in the right foot, suggesting a gender-related variation in small fiber integrity or sweat gland activity. Additionally, men had higher cardiovascular risk scores and LDL cholesterol levels than women, reinforcing the notion that sex-specific factors may influence both autonomic function and cardiometabolic risk profiles.

A key finding of our study was the strong inverse correlation between ESC in the lower extremities and cardiovascular risk scores, with the right foot ESC exhibiting the most robust association (r = −0.455, *p* < 0.001). These results highlight the potential role of sudomotor dysfunction as a marker of systemic autonomic dysregulation and underscore the clinical relevance of ESC in cardiovascular risk stratification. The observed relationship between reduced sweat gland activity and elevated cardiovascular burden may reflect underlying endothelial dysfunction, a shared pathway in both diabetic autonomic neuropathy and atherosclerosis. This is in agreement with earlier findings by Gatev et al. [13], who reported progressive declines in ESC values and increased asymmetry in individuals with diabetic foot complications. Also, Zhao et al. [14] found that reduced foot ESC was an independent risk factor for peripheral artery disease and could improve diagnostic accuracy when combined with the ankle–brachial index.

We also explored the relationship between ESC and glycemic control. Although a weak inverse correlation was found between HbA1c and left foot ESC (r = −0.150, *p* = 0.049), the significance suggests that chronic hyperglycemia may contribute to small fiber damage, even in the absence of overt symptoms. This finding aligns with previous reports that have identified chronic hyperglycemia as a contributing factor to autonomic and peripheral nerve dysfunction in individuals with diabetes. For example, D’Amato et al. [15] reported that lower ESC values were associated with worse cardiovascular autonomic neuropathy profiles in diabetic patients, a condition known to be exacerbated by prolonged hyperglycemia. Similarly, Lin et al. [16] found that ESC was significantly and inversely correlated with vibration perception threshold (VPT), another marker of diabetic neuropathy, and that ESC values were notably lower in individuals with poor metabolic control.

Our findings are also in agreement with Zhao et al. [14], who reported a significant association between ESC and cardiovascular/autonomic dysfunction in patients with T2DM. Although they did not specifically focus on HbA1c, the implication that microvascular and sudomotor impairment are linked to systemic metabolic dysregulation supports our interpretation that elevated HbA1c may indicate early nerve fiber injury, even in asymptomatic individuals.

Interestingly, a weak positive correlation was observed between BMI and left foot ESC. This unexpected result may reflect confounding factors such as increased skin hydration or regional variations in sweat gland responsiveness in individuals with higher BMI. However, this association did not extend to the hands or right foot and should, therefore, be interpreted with caution. However, since this correlation was limited to the left foot and not replicated in the right foot or hands, it is likely to be influenced by regional physiological variability or measurement artifacts. For instance, Lin et al. [16] did not report a significant association between ESC and BMI.

These results are partially supported by the study of Gatev et al. [13], who noted that ESC asymmetry and regional differences in sudomotor function could reflect early signs of diabetic foot complications. While they did not directly examine BMI, the presence of anatomical variability in ESC could provide a framework for understanding our findings. Additionally, as higher BMI is a known risk factor for both insulin resistance and altered thermoregulation, further research is needed to disentangle the physiological mechanisms that may underlie this association.

It is important to acknowledge that ESC measurements can be influenced by various external and physiological factors, including ambient temperature, skin hydration, and the use of topical agents. While all patients were instructed to avoid applying creams or lotions prior to testing and measurements were performed under standardized conditions, some interindividual variability may still have affected ESC readings. These factors represent potential limitations in the interpretation of sudomotor function, and future studies should aim to control and document these variables more rigorously.

Taken together, our findings reinforce the value of ESC as a marker for early neural compromise in diabetes while also highlighting the complexity of interpreting sudomotor function in the context of metabolic parameters, such as HbA1c and BMI. Future studies with larger cohorts and standardized protocols for hydration and skin temperature may help clarify these associations.

The diagnostic performance of ESC in detecting neuropathy was evaluated using ROC curve analysis. The AUC values ranged from 0.398 to 0.750, depending on the region assessed and the type of neuropathy. The highest diagnostic accuracy was achieved in the right foot for autonomic neuropathy, with an AUC of 0.750 (95% CI: 0.623–0.877, *p* < 0.001), demonstrating good discriminatory ability. This finding aligns with previous studies reporting that lower limb measurements, particularly in the feet, are more sensitive indicators of early small fiber dysfunction due to the length-dependent nature of diabetic neuropathy. For example, Lin et al. [16] reported that feet ESC values were significantly associated with vibration perception thresholds and that lower ESC values were independently predictive of DPN in a large population of patients with type 2 diabetes. Similarly, D’Amato et al. [15] found that combining ESC with the COMPASS 31 questionnaire improved sensitivity for detecting CAN to 100%, underscoring the importance of ESC in autonomic assessment.

Our results also support the well-documented concept that distal regions, particularly the feet, are more sensitive to early neuropathic changes due to the length-dependent degeneration of small fibers, as described in other studies [17,18,19,20,21]. This explains why feet ESC, especially from the right foot in our study, showed the strongest diagnostic value, while measurements from the upper extremities or non-dominant foot exhibited reduced performance.

The lower AUC values in certain anatomical regions may reflect interindividual variability in sudomotor function, asymmetrical progression of neuropathy, or confounding factors such as local skin temperature, hydration, or anatomical dominance. These factors have also been acknowledged in earlier literature and underline the importance of standardized measurement protocols when interpreting ESC results in clinical or research settings [22,23,24].

Our findings support the concept that electrochemical skin conductance (ESC) reflects small fiber autonomic nerve dysfunction, which is recognized as one of the earliest measurable indicators of diabetic complications. As ESC primarily assesses the function of unmyelinated sympathetic C-fibers innervating the sweat glands, its impairment may precede overt clinical symptoms of neuropathy. The significant inverse correlations observed between ESC and cardiovascular risk scores in our cohort—particularly in the lower extremities—further reinforce the potential of sudomotor dysfunction as an early marker of systemic autonomic and vascular impairment. Notably, these associations were evident even in asymptomatic patients, suggesting that ESC measurement could play a valuable role in early risk stratification and preclinical detection of diabetic complications. Also, these findings corroborate the growing body of evidence supporting the role of ESC—particularly from the lower limbs—as a practical, non-invasive tool for the early identification of diabetic neuropathy, with promising diagnostic performance when integrated into broader screening strategies. Conventional assessments of sudomotor function include quantitative sudomotor axon reflex testing, cardiovascular reflex testing, nerve conduction studies, and vibration perception thresholds [22,23]. These tests, while diagnostic, are time-intensive and require specialized equipment and trained staff, limiting their use in routine clinical practice. To address these challenges, we utilized SUDOSCAN, a rapid, non-invasive device that measures electrochemical skin conductance as a marker of small fiber and autonomic dysfunction. While SUDOSCAN offers a practical tool for early screening, we acknowledge that additional diagnostic methods may be required for comprehensive evaluation, especially in complex cases. While not all associations reached statistical significance, the overall trends suggest that ESC measurements via SUDOSCAN are clinically meaningful and non-invasively reflect systemic complication risk in patients with diabetes. Importantly, the device offers rapid, reproducible, and user-independent assessments, which could facilitate routine screening in outpatient settings.

## 5. Limitations and Clinical Implications

This study provides encouraging evidence supporting the use of SUDOSCAN-derived electrochemical skin conductance values as a non-invasive marker for assessing autonomic neuropathy and cardiovascular risk in diabetic patients. Additionally, SUDOSCAN cannot differentiate between etiologies of sudomotor dysfunction, and its specificity for diabetic neuropathy warrants further longitudinal validation. However, to strengthen the generalizability and clinical applicability of these findings, validation in larger and more diverse populations is warranted. Future research should aim to incorporate additional clinical and biochemical variables to enhance diagnostic and predictive accuracy. Furthermore, longitudinal studies are needed to assess the evolution of sudomotor dysfunction over time, potentially offering a valuable tool for tracking the progression of diabetic autonomic neuropathy and evaluating the efficacy of early therapeutic interventions. Continuous monitoring of ESC values and cardiovascular risk parameters may provide critical insights into the dynamics of systemic complications in diabetes and support more personalized care approaches. Integration of ESC measurements into routine diabetes care could enhance risk stratification and potentially guide individualized interventions.

## 6. Conclusions

Our findings support the use of SUDOSCAN as a non-invasive tool for identifying sudomotor dysfunction associated with diabetic peripheral and autonomic neuropathy. The strongest diagnostic performance was observed in the right foot ESC for autonomic neuropathy, with significant correlations to cardiovascular risk. These results highlight the potential role of ESC in early detection and risk stratification of diabetes-related complications.

## Figures and Tables

**Table 1:** Patient characteristics.

Variable	Men (Mean ± SD) (n = 156 Patients)	Women (Mean ± SD) (n = 132 Patients)	*p*-Value
**Demographic characteristics**			
**Age (years)**	59.3 ± 11.8	57.6 ± 12.6	0.218
**Rural area**	49.7%	50.3%	0.630
**Admission hemodynamics**			
**BMI (kg/m^2^)**	31.8 ± 15.9	33.2 ± 16.9	0.143
**Diabetes duration (years)**	9.8 ± 5.3	10.2 ± 4.6	0.643
**Systolic BP (mmHg)**	144.5 ± 23.7	141.5 ± 24.7	0.198
**Diastolic BP (mmHg)**	86.8 ± 56.1	83.4 ± 54.9	0.249
**HbA1c (%)**	8.5 ± 2.0	8.3 ± 1.9	0.368
**LDLc (mg/dL)**	134.6 ± 35.2	108.8 ± 36.1	0.042
**Cardiovascular risk score**	33.6 ± 11.9	30.5 ± 12.5	0.034
**Sudomotor function**			
**Right foot conductance (µS)**	76.5 ± 13.1	74.0 ± 13.5	0.041
**Left foot conductance (µS)**	75.9 ± 13.5	74.2 ± 13.9	0.072
**Right hand conductance (µS)**	65.2 ± 14.7	63.9 ± 14.9	0.095
**Left hand conductance (µS)**	66.3 ± 14.4	64.7 ± 14.8	0.112

BMI, body mass index; BP, blood pressure; HbA1c, glycated hemoglobin; LDLc, low density lipoprotein cholesterol.

## Data Availability

The data presented in this study are available on request from the corresponding author.

## Abbreviations

The following abbreviations are used in this manuscript:

AUC	Area under the curve
BMI	Body mass index
CAN	Cardiac autonomic neuropathy
DBP	Diastolic blood pressure
DFS	Diabetic foot syndrome
DN	Diabetic neuropathy
ESC	Electrochemical skin conductance
HbA1c	Glycated hemoglobin
LDLc	Low density lipoprotein cholesterol
PNP	Peripheral neuropathy
QSART	Quantitative sudomotor axon reflex testing
SBP	Systolic blood pressure
SD	Standard deviation
T2DM	Type 2 diabetes mellitus
T1DM	Type 1 diabetes mellitus
VPT	Vibration perception threshold
